# Supplementary material for: Asymmetric Synthesis of Quaternary Hydantoins via a Palladium-Catalyzed Aza-Heck Cyclization
Source: J Am Chem Soc. 2025 Nov 14;147(49):44692–8. doi: 10.1021/jacs.5c16022 (PMC12703750; doi:10.1021/jacs.5c16022)

D:\data\2024\DWatson_Temidayo_Idowu_197 02/18/25 16:24:47

DWatson_Temidayo_Idowu_197 #29 RT: 0.28 AV: 1 NL: 5.03E8
T: FTMS + p ESI Full ms [100.0000-1500.0000]

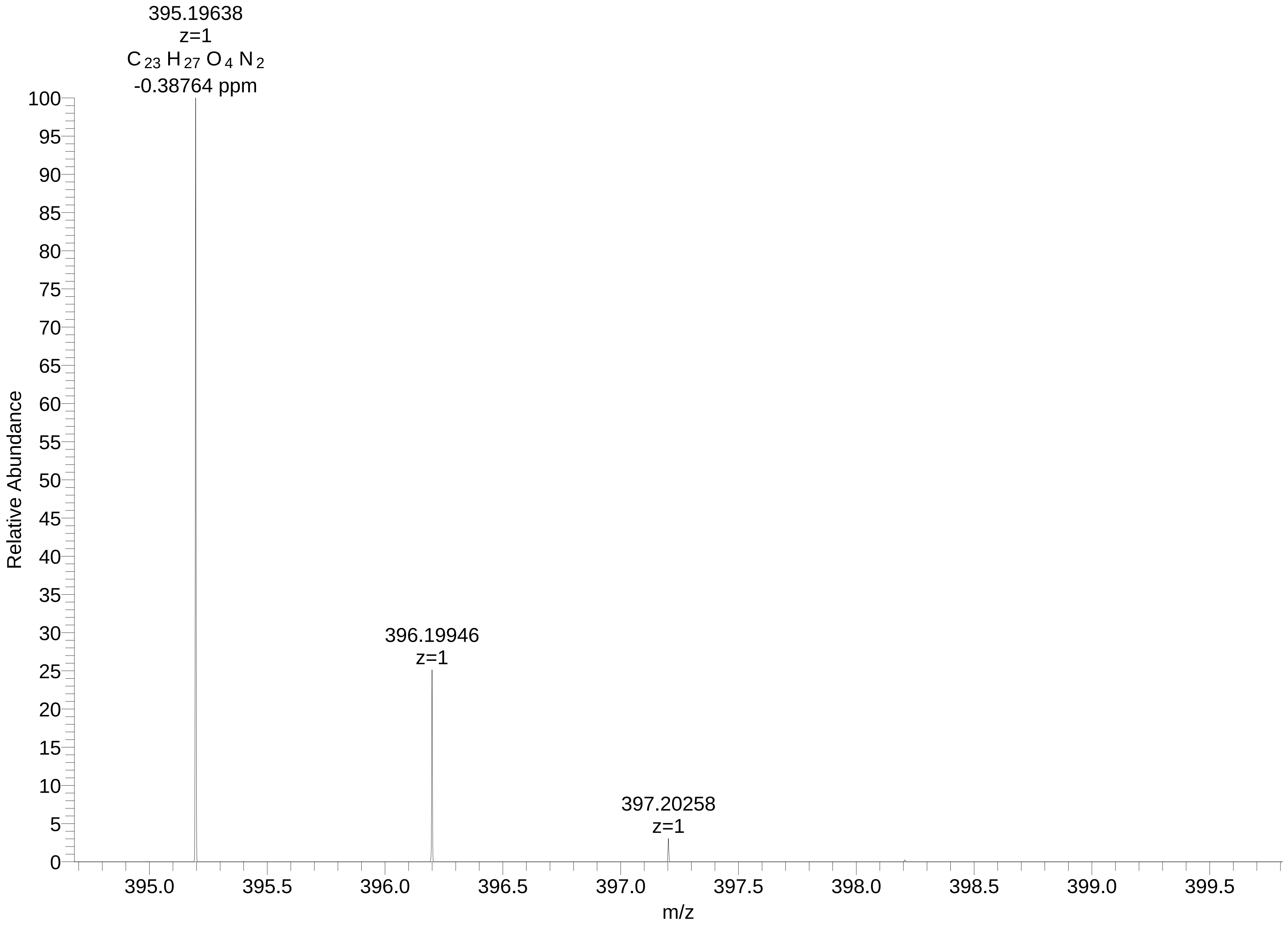

Supplement: Supplementary file 2 [file ja5c16022_si_002.zip › All NMR FID Files/S31/S31_AllNMR/HRMS/DWatson_TI_197.pdf]
